# Retraction: 3, 3′-Diindolylmethane Exhibits Antileukemic Activity *In Vitro* and *In Vivo* through a Akt-Dependent Process

**DOI:** 10.1371/journal.pone.0229164

**Published:** 2020-02-06

**Authors:** The PLOS ONE Editors

The University of Kentucky requested retraction of this article based on the findings of an institutional investigation. The institutional committee concluded that the article contains results that could not be validated with original data, and noted concerns about how experiments and results were documented in the laboratory’s records.

In our editorial review of this case, concerns were raised about the uniformity and lack of visible image details in the background areas of blot fragments reported in the western blot figures. In response to journal queries the authors provided cropped versions of the reported images with gray rather than white background and with improved visibility of faint bands in some cases. However, the images provided did not fully resolve the concerns about the integrity of these results.

In addition, similarities were noted between β-actin data reported in the following figure panels:

- Fig 1b, c, d
- Fig 3b, c, d
- Fig 4b, c
- Fig 4e, f
- Fig 5b, c

The authors commented that the experiments in each set of figure panels used the same samples and the same experimental conditions, and so the same control blots were relevant for the indicated panels.

In light of the above concerns and the recommendation by the University of Kentucky, the *PLOS ONE* Editors retract this article.

NG disagrees with the retraction and stands by the integrity of the data and the validity of the reported results. ZZ did not agree with retraction. The other authors either did not reply or could not be reached.
